# [^18^F]FDG PET/MRI in Endometrial Cancer: Prospective Evaluation of Preoperative Staging, Molecular Characterization and Prognostic Assessment

**DOI:** 10.3390/cancers18020280

**Published:** 2026-01-16

**Authors:** Carolina Bezzi, Gabriele Ironi, Tommaso Russo, Giorgio Candotti, Federico Fallanca, Carlotta Sabini, Ana Maria Samanes Gajate, Samuele Ghezzo, Alice Bergamini, Miriam Sant’Angelo, Luca Bocciolone, Giorgio Brembilla, Paola Scifo, GianLuca Taccagni, Onofrio Antonio Catalano, Giorgia Mangili, Massimo Candiani, Francesco De Cobelli, Arturo Chiti, Paola Mapelli, Maria Picchio

**Affiliations:** 1 Nuclear Medicine Department, IRCCS San Raffaele Scientific Institute, 20132 Milan, Italy; bezzi.carolina@hsr.it (C.B.); fallanca.federico@hsr.it (F.F.); samanesgajate.anamaria@hsr.it (A.M.S.G.); ghezzo.samuele@hsr.it (S.G.); scifo.paola@hsr.it (P.S.); chiti.arturo@hsr.it (A.C.); mapelli.paola@hsr.it (P.M.); 2 Radiology Department, IRCCS San Raffaele Scientific Institute, 20132 Milan, Italy; ironi.gabriele@hsr.it (G.I.); russo.tommaso1@hsr.it (T.R.); brembilla.giorgio@hsr.it (G.B.); decobelli.francesco@hsr.it (F.D.C.); 3 School of Medicine and Surgery, Vita-Salute San Raffaele University, 20132 Milan, Italy; bergamini.alice@hsr.it (A.B.); candiani.massimo@hsr.it (M.C.); 4 Unit of Obstetrics and Gynaecology, IRCCS San Raffaele Scientific Institute, 20132 Milan, Italy; candotti.giorgio@hsr.it (G.C.); sabini.carlotta@hsr.it (C.S.); bocciolone.luca@hsr.it (L.B.); mangili.giorgia@hsr.it (G.M.); 5 Pathology Unit, IRCCS San Raffaele Scientific Institute, 20132 Milan, Italy; santangelo.miriam@hsr.it (M.S.); taccagni.gianluca@hsr.it (G.T.); 6 Department of Radiology, Massachusetts General Hospital. Harvard Medical School, Boston, MA 02114, USA; ocatalano@mgh.harvard.edu

**Keywords:** [^18^F]FDG PET/MRI, endometrial cancer, staging, molecular characterization, recurrence prediction

## Abstract

**Background/Objectives:** Early and accurate characterization of endometrial cancer (EC) is crucial for patient management, but current imaging modalities lack in diagnostic accuracy and ability to assess molecular profiles. The aim of this study is to evaluate hybrid [^18^F]FDG PET/MRI’s diagnostic accuracy in EC staging and role in predicting tumor aggressiveness, molecular characterization, and recurrence. **Methods**: A prospective study (ClinicalTrials.gov, ID:NCT04212910) evaluating EC patients undergoing [^18^F]FDG PET/MRI before surgery (2018–2024). Histology, immunohistochemistry, and patients’ follow-up (mean FU time: 3.13y) were used as the reference standard. [^18^F]FDG PET/MRI, PET only, and MRI only were independently reviewed to assess the diagnostic accuracy (ACC), sensitivity (SN), specificity (SP), and positive/negative predictive value (PPV, NPV). Imaging parameters were extracted from [^18^F]FDG PET and pcT1w, T2w, DWI, and DCE MRI. Spearman’s correlations, Fisher’s exact test, ROC-AUC analysis, Kaplan–Meier survival curves, log-rank tests and Cox proportional hazards models were applied. **Results**: Eighty participants with primary EC (median age 63 ± 12 years) were enrolled, with 17% showing LN involvement. [^18^F]FDG PET/MRI provided ACC = 98.75%, SN = 98.75%, and PPV = 100% for primary tumor detection, and ACC = 92.41%, SN = 84.62%, SP = 93.94%, PPV = 73.33%, and NPV = 96.88% for LN detection. PET/MRI parameters predicted LN involvement (AUC = 79.49%), deep myometrial invasion (79.78%), lymphovascular space invasion (82.00%), p53abn (71.47%), MMRd (74.51%), relapse (82.00%), and postoperative administration of adjuvant therapy (79.64%). Patients with a tumor cranio-caudal diameter ≥ 43 mm and MTV ≥ 13.5 cm^3^ showed increased probabilities of recurrence (*p* < 0.001). **Conclusions**: [^18^F]FDG PET/MR showed exceptional accuracy in EC primary tumor and LN detection. Derived parameters demonstrated potential ability in defining features of aggressiveness, molecular alterations, and tumor recurrence.

## Simple Summary

This prospective study evaluated the value of hybrid [^18^F]FDG PET/MRI in supporting risk stratification of endometrial cancer (EC). The cohort included 80 women with newly diagnosed EC, who underwent [^18^F]FDG PET/MRI before surgery and were followed for recurrence. PET/MRI showed excellent diagnostic accuracy for both primary tumor detection and lymph node assessment. Multiparametric PET/MRI features were analyzed and predicted several indicators of tumor aggressiveness such as the molecular alterations of p53 abnormalities and MMR deficiency recently introduced in the updated FIGO staging system and the clinical indicators of relapse risk and need for adjuvant therapy. Overall, PET/MRI demonstrated meaningful potential for predicting tumor behavior and improving risk stratification and personalized treatment planning in endometrial cancer patients.

## 1. Introduction

Endometrial cancer (EC) ranks as the sixth most common cancer among women worldwide [1]. It is broadly classified into two types: type I, accounting for 85% of cases, predominantly includes grades 1 and 2 endometrioid adenocarcinomas (EEC); type II, which includes aggressive histological types, such as grade 3 EEC, typically associated with poorer prognoses [2]. Treatment includes surgery, radiation, chemotherapy, and hormonal therapy, with radical surgery and lymphadenectomy recommended for high-risk cases. However, clinical outcomes can differ even in patients with similar histological profiles, and the accurate selection of patients who might benefit from these treatments remains challenging.

Traditional prognostic factors include Federation of Gynecology and Obstetrics (FIGO) stage, histological type, depth of myometrial invasion (MI), lymph node (LN) metastases, infiltration pattern, and lymphovascular space invasion (LVSI) [3,4,5]. Moreover, many advancements in molecular profiling, driven by The Cancer Genome Atlas (TCGA) [6], have occurred since the 2009 FIGO staging, including the identification of four distinct EC molecular subtypes with significant prognostic value: POLE ultramutated, microsatellite instability hypermutated, copy number low, and copy number high subtypes. Therefore, an updated FIGO staging system was proposed in 2023 [7], in which molecular classification testing (POLE sequencing, MMR–immunohistochemistry (HIC), and p53-HIC) is strongly encouraged. However, these histological and molecular factors can only be assessed after surgery, with some factors available preoperatively through biopsy, which cannot fully capture tumor heterogeneity.

Imaging plays a crucial role in the assessment of EC. Transvaginal ultrasound, Magnetic Resonance Imaging (MRI), and Computed Tomography (CT) provide relevant morphological information. [^18^F]fluoro-deoxyglucose ([^18^F]FDG) Positron Emission Tomography (PET)/ CT is included in clinical guidelines for EC management and provides metabolic information, as shown in [8,9]. The introduction of hybrid PET/MRI scanners allows full integration of anatomical and metabolic information, reducing radiation exposure and providing multifunctional imaging biomarkers with relevant information on tumor behavior and underlying biological processes [10,11,12,13].

In the present study, we investigated the diagnostic accuracy of hybrid [^18^F]FDG PET/MRI for preoperative staging of endometrial cancer (EC), assessing both primary tumor and lymph node involvement. Additionally, we investigated its predictive role in identifying histological features of aggressiveness, molecular profiles, and recurrence, with the ultimate goal of enhancing treatment personalization.

## 2. Materials and Methods

### 2.1. Participants

**Figure 1:**
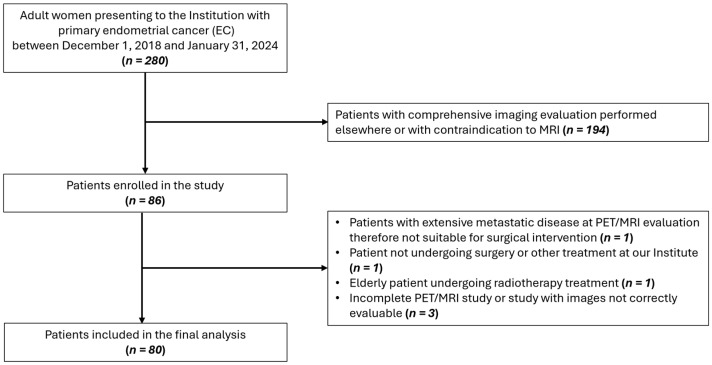
Flow diagram showing participant selection details.

In this prospective study (ClinicalTrials.gov ID: NCT04212910), 80 patients aged ≥18, with histological diagnosis of EC, no contraindication to preoperative [^18^F]FDG PET/MRI, and suitability for surgical intervention were enrolled between December 2018 and January 2024. A flow diagram showing participant selection is displayed in Figure 1. The study received approval from the Institution’s Ethics Committee and informed consent was obtained from all participants. Instrumental and clinical follow-up data were collected until February 2025. All procedures were carried out in accordance with the Declaration of Helsinki (1964) and its later amendments.

### 2.2. [^18^F]FDG PET/MRI Protocol

All scans were performed using a fully hybrid 3 T PET/MR system (SIGNA PET/MR; General Electric Healthcare, Waukesha, WI, USA), as described in [13]. Specifically, a simultaneous whole-body (WB) PET/MR scan was performed approximately 60 min (range 60–90) after the intravenous administration of [^18^F]FDG, with the dosage administered following the EANM procedure guidelines for tumor imaging [14]. Four or five FOVs (4 min each) were acquired depending on patients’ height. Simultaneously, MRAC and LAVA-Flex images were acquired for AC correction and anatomical localization, respectively. Following the acquisition of the WB scan and a brief break, a dedicated single PET/MRI-FOV centered on the pelvic region was performed after the intramuscular injection of 40 mg of hyoscine butyl bromide (Buscopan; Boehringer Ingelheim, Ingelheim am Rhein, Germany) to minimize artifacts related to bowel motion. Parallelly, a full set of diagnostic MR sequences were acquired, including the following: (1) a large FOV axial T2 imaging of the pelvis; (2) high-resolution small FOV sagittal, axial, and coronal T2-weighted FSE PROPELLER; (3) a small FOV axial DWI sequence covering the primary tumor (b values, 0–200–600–1000 s/mm^2^); (4) a dynamic contrast-enhanced (DCE) MR study using a LAVA sequence with a temporal resolution of 4 s. Sequential images were acquired before and up to 5 min after intravenous injection of 0.1 mmol/kg of gadolinium chelate (Gadovist; Bayer, Milan, Italy). Finally, a small FOV high-resolution 3D fat-suppressed T1w sequence of the uterus was performed. The total acquisition time per participant was approximately 1 h. PET images were corrected for attenuation using the MRAC sequences simultaneously acquired, with the image reconstruction applying a Bayesian penalized likelihood algorithm (Q.Clear-GE).

### 2.3. [^18^F]FDG PET/MRI Analysis

Qualitative and quantitative analysis was performed following current guidelines [15,16] on the Advantage Workstation (ADW Dexus Version 4.7), as described in [13]. Two Nuclear Medicine and two Radiologists separately evaluated the PET and MRI scans, respectively, to provide single modality assessment of the primary tumor and LNs, while being blinded to the results of the histopathologic examinations. A consensus was reached at the conclusion of the assessment to produce a combined PET/MRI evaluation, where primary tumor and LNs findings were classified as positive if deemed suspicious on either PET or MRI evaluation. On MR scans, tumor infiltration in the myometrium, serosa, parametria, and cervical stroma was assessed. LNs were regionally classified as pelvic (obturator and iliac) and abdominal (para-aortic and intercavo-aortic), with the latter being excluded from MRI region-based analysis due to its limited field of view. Post-surgical histopathology was used as reference standard. Max and mean standardized uptake value (SUVmax, SUVmean), metabolic tumor volume (MTV), and total lesion glycolysis (TLG) were calculated within the metabolically active tumor volume defined by a thresholding-based approach (41% of SUVmax), following EANM procedure guidelines [14]. The following MRI dimensional parameters were assessed on post-contrast T1w (pcT1w), T2w, and DWI sequences: tumor antero-posterior, latero-lateral, and cranio-caudal diameters (Size_AP, Size_LL, Size_CC), volume index (VI) resulting from the multiplication of these three diameters, total tumor volume (TTV), total uterine volume (TUV), and tumor volume ratio (TVR = TTV/TUV). Apparent diffusion coefficients (ADCmean, ADCmin, ADCmax) derived from the DWI sequence were noted. DCE-MRI analyses were performed using a dedicated tool (GenIQ) on Advantage Workstation, based on the Toft model [17]. A two-dimensional ROI was manually drawn on perfusion maps on the slice where the tumor had the greatest extension, attempting to exclude healthy tissue and areas of internal necrosis. In this way, the following DCE-MRI parameters were extracted: transfer constant (Ktrans), efflux rate (Kep), extravascular extracellular volume (Ve), contrast–enhancement ratio (CER), integral area under the curve (IAUC), and maximum slope of increase (maxSLOPE). The definitions of the extracted parameters and their calculations are described in [13].

### 2.4. Surgery and Histopathological Analysis

Surgical procedures and adjuvant therapy were performed according to standard clinical guidelines [8]. Surgical intervention involved total open or laparoscopic hysterectomy, bilateral salpingo-oophorectomy, and peritoneal washing. At histology, tumor histotype, grade, myometrial infiltration, serosal, parametrial and cervical stromal invasion, and LVSI were assessed, blinded to imaging data. For nodal staging, histopathological findings following pelvic/para-aortic lymphadenectomy or sentinel lymph node dissection were considered as the reference standard. Staging was determined in accordance with the FIGO classification of endometrial tumors [18]. For the immunohistochemical analyses, slides of formalin-fixed, paraffin-embedded EC tissues were stained using specific monoclonal antibodies: Estrogen receptor-ER (CONFIRM anti-estrogen receptor SP1), Progesterone receptor-PGR (CONFIRM anti-progesterone receptor 1E2), p53 (CONFIRM anti-p53 DO7), MLH1 (BOND ready-to-use primary antibody ES05), MSH2 (BOND ready-to-use primary antibody 79H11), MSH6 (BOND ready-to-use primary antibody EP49), and PMS2 (BOND ready-to-use primary antibody EP51). The ER, PGR, and p53 markers were stained using the automated BenchMark ULTRA system (Roche Diagnostics, Monza, Italy); the MMR markers were stained with the automated BOND-III system (Leica Biosystems, Milan, Italy). ER and PGR expression were quantified as percentages. p53 positivity was assessed to determine whether the expression was wild-type or indicative of mutations (p53abn). p53abn expression was defined as either overexpression of p53 in >80% of tumor cells or complete absence of nuclear staining in tumor cells. Mismatch repair deficiency (MMRd) was identified by the complete absence of nuclear expression of at least one MMR protein (MLH1, MSH2, MSH6, or PMS2) in carcinoma cells, with stromal and/or lymphocytic cells used as internal controls [19].

### 2.5. Statistical Analysis

Statistical analyses were performed with Python 3.12.2. Continuous variables were expressed as mean ± standard deviation (SD) or median and interquartile range (IQR); discrete variables were summarized as frequencies and percentages. For qualitative analysis, differences in the modalities of SN and SP were assessed using McNemar’s test, while agreement between imaging and histology was evaluated with Cohen’s kappa (κ). Relationships among quantitative parameters were assessed using Spearman’s correlation; associations between parameters and outcomes were assessed using Fisher’s exact test for dichotomous data, and receiver operating characteristics (ROC) curve analysis for continuous data, with area under the curve (AUC) values interpreted as follows: 50–60% = fail, 60–70% = poor, 70–80% = fair, >80% = good. Optimal cut-offs for significant AUCs were determined via Youden’s J index. Tumor relapse prognosis was assessed with Kaplan–Meier curves (using cut-off values identified in the ROC analysis) and log-rank tests, with univariate Cox proportional hazards models quantifying relapse risk and performance being assessed by the C-index. Benjamini–Hochberg correction was applied for multiple comparisons. Significance was set at *p* < 0.05.

## 3. Results

### 3.1. Participants and EC Characteristics

The study cohort included 80 participants with a mean age of 63 years ±12 (SD). Surgery was performed after a mean of 22 ± 15 days following the PET/MR examination, without any change in medical therapy between imaging and resection. Participants’ demographics and tumor characteristics are presented in Table 1.

### 3.2. [^18^F]FDG PET/MRI Diagnostic Accuracy

For primary tumor detection, focal pathological [^18^F]FDG uptake was detected in 78/80 participants (ACC = 97.5%, SN = 97.5%, PPV = 100%); MR accurately identified 79/80 primary tumors (ACC = 98.75%, SN = 98.75%, PPV = 100%). [^18^F]FDG PET/MRI metrics were ACC = 98.75%, SN = 98.75%, PPV = 100%, with only one participant whose tumor was not detected (endometrioid G2 tumor with a max diameter of 1.5 cm). No significant difference between PET and MR sensitivity was observed (*p* > 0.99).

**Figure 2:**
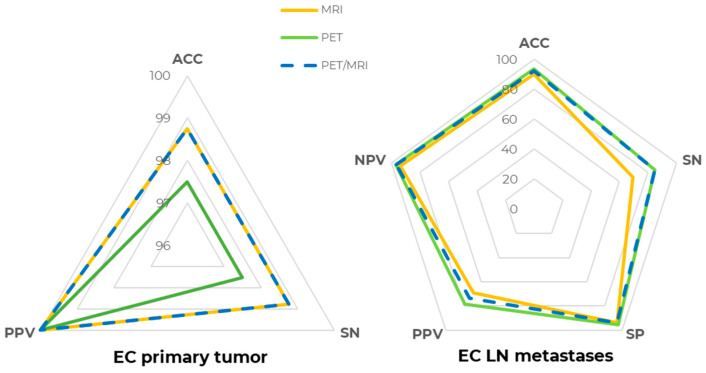
[^18^F]FDG PET/MRI diagnostic performance at the patient level. Spider plot summarizing [^18^F]FDG PET (green line), MR (yellow line), and [^18^F]FDG PET/MR (dashed blue line) diagnostic performance in staging EC primary tumor (**right**) and lymph node (LN) metastases (**left**). ACC: accuracy, SN: sensitivity, SP: specificity, PPV: positive predictive value, NPV: negative predictive value.

For LN metastases detection, [^18^F]FDG PET ACC at the patent-level was 93.67% (SN = 84.62%, SP = 95.45%, PPV = 78.57%, NPV = 96.92%), whereas MR, evaluating pelvic lymph nodes only due to its limited field of view, had a slightly lower performance, with an ACC of 89.87% (SN = 69.23%, SP = 93.94%, PPV = 69.23%, NPV = 93.94%), even though the difference in both SN and SP was not statistically significant (*p* > 0.05). Precisely, two patients with abdominal lymph node metastases were correctly detected by PET but could not be assessed by MRI, contributing to the lower MRI sensitivity. [^18^F]FDG PET/MRI metrics were ACC = 92.41%, SN = 84.62%, SP = 93.94%, PPV = 73.33%, and NPV = 96.88%, which were slightly lower than [^18^F]FDG PET alone due to the one false positive lesion detected by MRI. Patient-level [^18^F]FDG PET, MR, and [^18^F]FDG PET/MR detection metrics are compared and shown in Figure 2.

**Figure 3:**
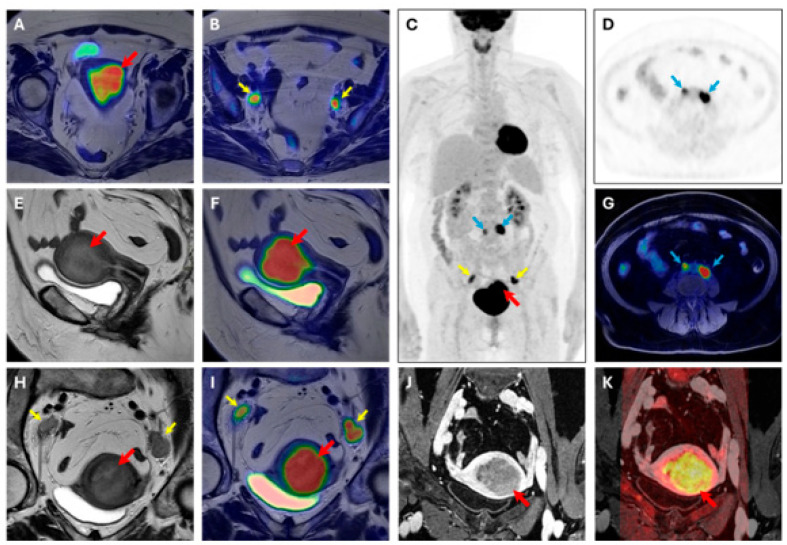
[^18^F]FDG PET/MRI scan. Image of a 63-year-old woman with endometrial cancer (stage IIIC, endometrioid grade 2, LVSI positive, p53 preserved, MMRd, myometrial invasion > 50%). (**A**,**B**) axial large field-of-view T2W-PET fusion image, (**C**) coronal whole body PET, (**D**) axial PET image, (**E**) sagittal T2W image, (**F**) sagittal T2W-PET fused image, (**G**) axial T1 fat suppression–PET fused image, (**H**) axial oblique T2W image, (**I**) axial oblique T2W-PET fused image, (**J**) axial oblique T1 post-contrast agent administration, (**K**) axial oblique T1 post-contrast agent administration-DWI b1000 fused image. Volume index: 114 cc; TTV: 59 cc; TVR: 68%; ADCmean: 1.19 × 10^−3^ mm^2^/s; ADCmin: 0.253 × 10^−3^ mm^2^/s. PET parameters of endometrial cancer SUVmax 19.42; SUVmean:13.27; MTV: 34.15; TLG: 453.2. Red arrow: primary EC; yellow arrow: pelvic lymph node metastases; blue arrow: abdominal lymph node metastases.

At the region-based level, pelvic LNs were detected with an ACC of 93.51% (SN = 81.82%, SP = 95.45%, PPV = 75.00%, and NPV = 96.92%) by [^18^F]FDG PET, an ACC of 92.21% (SN = 81.82%, SP = 93.94%, PPV = 69.23%, and NPV = 96.87%) by MRI, and an ACC of 92.21% (SN = 81.82%, SP = 93.94%, PPV = 69.23%, and NPV = 96.87%) by [^18^F]FDG PET/MRI. For abdominal LNs, an ACC of 95.65% (SN = 100.00%, SP = 95.45%, PPV = 50.00%, and NPV = 100.00%) was observed for [^18^F]FDG PET, and thus [^18^F]FDG PET/MRI was the region that was excluded from the MRI’s field of view. For all LNs diagnosed as pathological by [^18^F]FDG PET but later confirmed as false positives, histopathological analysis confirmed a chronic reactive lymphadenitis, partly of the hyperplastic type. One metastasis was also found in the inguinal region, and both [^18^F]FDG PET and MRI correctly detected it. MRI also demonstrated substantial and moderate agreement (κ = 0.65, κ = 0.54) in the detection of myometrial and cervical stroma invasion, respectively. However, none of the tumors characterized by parametria infiltration were detected, and only 1/5 tumors infiltrating the serosa were identified. For LNs detection, [^18^F]FDG PET demonstrated an almost perfect agreement (κ = 0.82), while MRI showed a substantial one (κ = 0.60). A representative case of [^18^F]FDG PET/MR scan is displayed in Figure 3.

### 3.3. [^18^F]FDG PET/MR Parameters’ Evaluation

Correlations among all investigated parameters are displayed in Supplemental Figure S1. A strong positive correlation was observed among all imaging parameters describing tumor size, regardless of the modality/sequence. Strong and moderate correlations were also found within MR parameters describing tumor perfusion and within those describing diffusion, while no significant correlation was observed between perfusion and diffusion parameters. Associations between [^18^F]FDG PET/MR-detected deep MI, cervical stroma invasion, and LN metastases with investigated outcomes are summarized in Table 2. Precisely, MRI-detected myometrial invasion was significantly associated with tumor recurrence, with patients showing invasion on MRI having 13.7 times higher odds compared to those without signs of invasion (*p* = 0.02).

**Figure 4:**
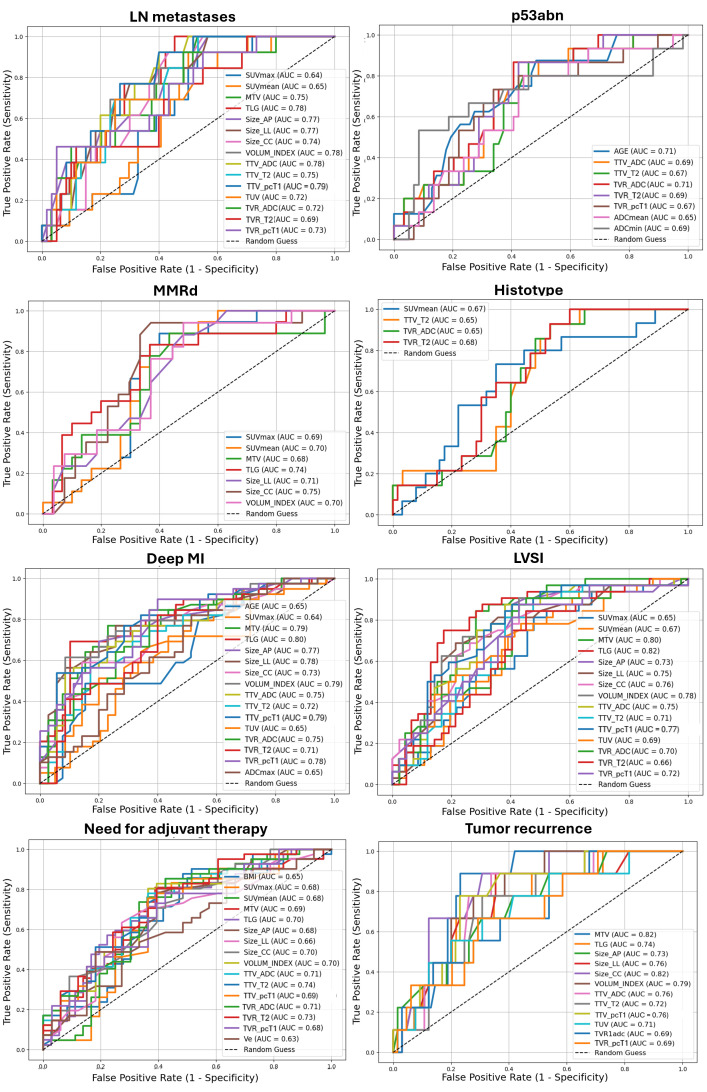
Receiver operating characteristic (ROC) curves with AUC values of [^18^F]FDG PET/MR and clinical (age, BMI) parameters in predicting different outcomes of EC aggressiveness. Only statistically significant results were displayed. LN: lymph nodes; p53abn: abnormal p53; MMRd: DNA mismatch repair deficiency; LVSI: lymphovascular space invasion; MI: myometrial invasion. Volumetric parameters are expressed in mL or cm^3^; TVR is expressed as percentage (%); ADC values are expressed as 10^−6^ mm^2^/s. Size_AP = antero-posterior diameter, Size_LL = latero-lateral diameter, Size_CC = cranio-caudal diameter, TTV = total tumor volume, TUV = total uterine volume, TVR = tumor volume ratio, ADC = apparent diffusion coefficient, Ktrans = transfer constant, Kep = efflux rate, Ve = extravascular extracellular volume, CER = contrast–enhancement ratio, maxSLOPE = maximum slope of increase, SUV = standardized uptake value, MTV = metabolic tumor volume, TLG = total lesion glycolysis.

Statistically significant AUCs for each outcome are compared and displayed in Figure 4, and summarized, together with 95% CI, optimal cut-offs, and the respective SN and SP in Supplemental Table S1. [^18^F]FDG PET/MR parameters demonstrated, at best, an AUC = 79.49%, SN = 92.30%, and SP = 60.00% (best parameter: TTV calculated on pcT1w MRI, cut-off: 9.00 mm) in the prediction of LN metastases. Additionally, [^18^F]FDG PET/MR parameters demonstrated higher performance in the assessment of deep MI compared to qualitative analysis (best parameter: TLG, cut-off: 119.40 cm^3^, with AUC = 79.78%, SN = 69.23%, SP = 89.74%), and good performance in the characterization of LVSI (best parameter: TLG, cut-off: 114.20 cm^3^, with AUC = 82.00%, SN = 75.00%, SP = 82.61%). Less accurate predictions were found for EC histotype (best parameter: TVR derived from T2w MRI, cut-off: 9.61 mm, with AUC = 67.86%, SN = 100.00%, SP = 40.00%) and infiltration pattern (best parameter: SUVmean, cut-off: 14.14, with AUC = 65.00%, SN = 68.42%, SP = 69.23%).

**Figure 5:**
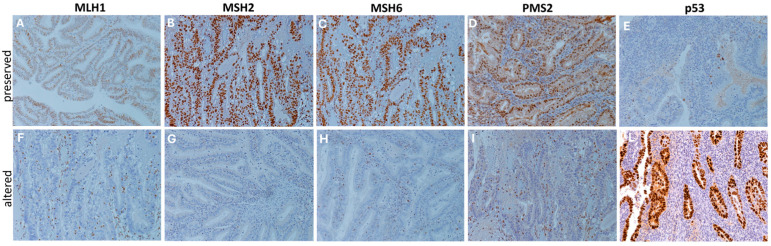
DNA mismatch repair and p53 proteins immunohistochemical staining. Representative immunohistochemical stainings (20×) showing cases of preserved (**A**–**E**) and altered (**F**–**L**) expression patterns of DNA mismatch repair proteins (MLH1, MSH2, MSH6, and PMS2) and p53 proteins. Protein alteration was identified by the complete absence of nuclear expression in carcinoma cells, with stromal and/or lymphocytic cells used as internal controls, following established guidelines [20]. Staining was performed using specific monoclonal antibodies: p53 (CONFIRM anti-p53 DO7), MLH1 (BOND ready-to-use primary antibody ES05), MSH2 (BOND ready-to-use primary antibody 79H11), MSH6 (BOND ready-to-use primary antibody EP49), and PMS2 (BOND ready-to-use primary antibody EP51).

According to macromolecular characterization, [^18^F]FDG PET/MRI parameters demonstrated fair predictions for MMRd (best parameter: cranio-caudal diameter measured on pcT1w MRI, cut-off: 30 mm, with AUC = 74.51%, SN = 94.12%, SP = 62.96%) and p53abn (best parameter: TVR derived from ADC maps, cut-off: 17.65 mm, with AUC = 71.47%, SN = 86.67%, SP = 59.32%). Notably, participants’ age provided comparable predictions for p53abn, with AUC = 71.47%, SN = 87.50%, SP = 51.61% using a cut-off of 61 years. Two representative cases of MMRd and p53 immunohistochemical images are displayed in Figure 5. Finally, regarding follow-up data, good and fair predictions were obtained for relapse (best parameter: MTV, cut-off: 13.5 cm^3^, with AUC = 82.50%, SN = 87.50%, SP = 78.57%) and postoperative administration of adjuvant therapy (best parameter: TTV calculated from T2w MRI, cut-off: 7 mm, with AUC = 73.50%, SN = 85.36%, SP = 57.57%), respectively.

**Figure 6:**
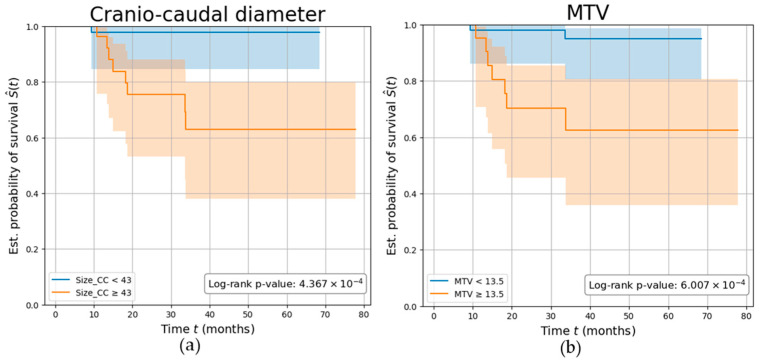
Parameters’ ability in predicting tumor recurrence. Kaplan–Meier curves showing the survival of EC participants stratified by (**a**) tumor’s cranio-caudal diameter measured on MRI (in mm), (**b**) tumor’s metabolic tumor volume (MTV) measured on PET (in cm^3^). The log-rank test *p*-value was calculated to compare the curves between the two groups.

Overall, patients were followed up for a mean (SD) of 3.13 (1.6) years, during which nine participants (11.25%) experienced disease recurrence. Parameters demonstrating the highest AUC among the PET (MTV) and MRI (Size_CC) values were independently investigated. Both parameters demonstrated a strong association with the risk of tumor relapse, with participants characterized by a Size_CC ≥ 43 mm and MTV ≥ 13.5 cm^3^ showing a significantly reduced disease-free survival (log-rank *p*-values *p* < 0.001), with a C-index of 0.77 and 0.76, respectively. Kaplan–Meier survival curves are shown in Figure 6.

## 4. Discussion

Accurate risk stratification and tumor staging are crucial for determining the optimal treatment strategy of EC patients, especially for younger patients of reproductive age who may benefit from fertility-sparing approaches. However, many features of tumor aggressiveness can only be assessed after surgery or through biopsy, which may not capture tumor heterogeneity. Moreover, patients with similar histological characteristics can present variable clinical outcomes.

This prospective study aimed to assess the diagnostic and prognostic value of fully hybrid [^18^F]FDG PET/MRI in endometrial cancer staging, specifically in predicting tumor aggressiveness, molecular characteristics, and tumor relapse. Our findings indicate that [^18^F]FDG PET/MRI accurately identified primary tumors (ACC = 98.75%, SN = 98.75%, PPV = 100%) and LN involvement (ACC = 92.41%, SN = 84.62%, SP = 93.94%, PPV = 73.33%, and NPV = 96.88%). Notably, the accuracy in identifying primary tumors was primarily achieved by MRI alone, which even slightly surpassed the performance of [^18^F]FDG PET/MR. Conversely, considering LNs involvement, PET alone outperformed [^18^F]FDG PET/MR. These findings confirm the value of [^18^F]FDG PET and MRI as standalone imaging modalities for primary tumors’ characterization and LN assessment, respectively. In centers that have access to hybrid PET/MR technology, this imaging tool represents the optimal choice, providing excellent preoperative staging, reducing the need for multiple tests, saving time, and reducing patient exposure to radiation from PET/CT. However, in centers where hybrid [^18^F]FDG PET/MR is not available, the combined use of [^18^F]FDG PET/CT and MRI still remains an effective and accurate approach for EC staging.

[^18^F]FDG PET/MRI parameters successfully predicted several features of tumor aggressiveness (AUCs of 79.49% for LN metastasis, 79.78% for deep MI, and 82.00% for LVSI), as well as key immunohistochemical molecular markers fundamental to the new molecular classification of EC patients (AUCs of 71.47% and 74.51% for p53abn and MMRd, respectively) and tumor relapse (AUC = 82.00%). Based on these results, [^18^F]FDG PET/MRI holds promise for early, non-invasive characterization of EC patients, offering potential advantages in patient management.

Our study’s metrics for primary tumor detection and LN involvement corroborate previous literature findings while highlighting the distinct contributions of PET and MRI modalities. Tsuyoshi et al. [20] found similar diagnostic accuracy in a study of 36 EC patients, where [^18^F]FDG PET/MRI demonstrated similar accuracy to conventional imaging and exceeded ceCT in sensitivity for regional nodal metastasis. Bian et al. [21], in a cohort of 81 patients, reported PET/MRI superior sensitivity and specificity for LN metastasis (95.5% vs. 86.5%, *p* < 0.005) compared to PET/CT.

Our analysis highlighted strong correlations among the different parameters describing tumor size, regardless of the imaging modality or sequence. This correlation, also observed in previous research, emphasizes the metabolic component’s role in EC growth and progression, emphasizing the potential of metabolic-targeting agents such as metformin as auxiliary treatments, especially in cases resistant to standard chemotherapy [22,23]. Parameters describing tumor size showed good predictive value for several features of aggressiveness, aligning with previous studies [24,25] and Mayo criteria for risk assessment [26]. Despite these findings, the FIGO staging system includes tumor size as a standard measurement for cervical cancer, but not for EC [27].

Regarding the investigation on the relationship between [^18^F]FDG PET/MR imaging parameters and the recent EC molecular characterization, MMRd correlated with SUVmax and TLG, along with MRI-derived cranio-caudal diameter and volume index. This new finding aligns with studies indicating that the immune microenvironment in MMRd subtypes is characterized by a higher lymphocyte infiltration and thus affected by higher glucose uptake [28,29,30]. For p53abn, associations were instead found with ADC-based tumor volume ratios and age. This could help tailor treatment strategies, since recent studies have shown improved survival with chemotherapy compared to radiation alone for p53abn EC [31]. According to literature, Tian et al. [32] demonstrated that multimodal MRI, including DWI, can distinguish p53abn from p53 wild-type EC, while Zhang et al. [33] found that ADC values were significantly higher in p53abn EC than in other subtypes.

Our study also demonstrated associations between [^18^F]FDG PET/MRI parameters and recurrence risk, with participants characterized by a tumor’s cranio-caudal size ≥ 43 mm or MTV ≥ 13.5 cm^3^ experiencing significantly reduced disease-free survival. Moreover, patients showing myometrial invasion on the MRI had 13.7 times higher odds of tumor relapse compared to those without signs of invasion (*p* = 0.02).

This study has some limitations. First, it should be noted that MRI lymph node evaluation in our study was limited to pelvic regions due to the MRI restricted field of view, which excluded abdominal lymph nodes from assessment, and this anatomical coverage limitation should be considered when interpreting the comparative performance of the two modalities for LNs detection. Furthermore, our sample showed limited number of events and class imbalances, particularly in LNs and recurrence analysis, although remaining consistent with general population incidence. Additionally, data for molecular profiling were incomplete and POLE mutational status was not assessed. Finally, the cut-off values used for survival analysis require external validation before clinical application, as they were derived from ROC analysis within our cohort and may thus overestimate prognostic performance. Due to these limitations, the presented findings should be considered exploratory and hypothesis-generating, and future studies with larger sample sizes, comprehensive molecular profiling including, and independent external validation cohorts will be needed to confirm these associations and establish clinically applicable thresholds.

In conclusion, [^18^F]FDG PET/MRI demonstrated excellent diagnostic and prognostic performance for EC staging and recurrence prediction, with PET/MRI parameters offering insight into tumor aggressiveness and molecular characteristics. Future studies should focus on expanding cohort sizes to validate these findings and exploring tailored therapeutic interventions and follow-up protocols.

## 5. Conclusions

[^18^F]FDG PET/MRI demonstrated high diagnostic accuracy in the preoperative staging of EC, effectively identifying both primary tumor extent and lymph node metastases. Additionally, [^18^F]FDG PET/MRI-derived imaging parameters showed association with histological features of tumor aggressiveness, molecular markers (including p53abn and MMRd), and risk of tumor relapse. These findings underscore the potential of this hybrid technique to support optimal patient risk stratification and personalized treatment planning, even though future validation is needed before clinical application.

## Figures and Tables

**Table 1:** Demographic and baseline clinical characteristics of the participants.

Characteristic	Value
Age, years	63 (11.8)
FIGO stage	
IA	32 (40.00%)
IB	25 (31.25%)
II	3 (3.75%)
IIIA	4 (5.00%)
IIIB	1 (1.25%)
IIIC1	9 (11.25%)
IIIC2	2 (2.50%)
IVB	4 (5.00%)
Grade	
1	9 (11.20%)
2	45 (56.20%)
3	26 (32.50%)
Histology	
Endometroid	64 (80.00%)
Serous	6 (7.50%)
Mixed serous/endometroid	1 (1.20%)
Mixed clear cells/serous	2 (2.50%)
Other (MMT/squamocellular)	7 (8.70%)
Infiltration pattern	
Infiltrative	16 (20.00%)
Expansive	51 (63.70%)
Infiltrative and Expansive	3 (3.70%)
MELF	1 (1.25%)
No infiltration	9 (11.20%)
MMRd	
yes	18 (22.50%)
no	31 (38.75%)
unavailable	31 (38.75%)
Lymph node metastases	
yes	13 (16.70%)
no	65 (81.25%)
unavailable	2 (2.50%)
p53	
abnormal (abn)	17 (21.25%)
wild-type	62 (77.50%)
unavailable	1 (1.20%)
Lymph vascular space invasion	
yes	31 (38.70%)
no	49 (61.30%)
Myometrial invasion	
yes	38 (47.50%)
no	42 (52.50%)
Serosa invasion	
yes	5 (6.25%)
no	73 (91.25%)
unavailable	2 (2.50%)
Parametria invasion	
yes	3 (3.75%)
no	76 (95.00%)
unavailable	1 (1.25%)
Cervical stroma invasion	
yes	16 (20.00%)
no	64 (80.00%)
Adjuvant therapy administration *	
yes	43 (53.70%)
no	37 (46.20%)
Tumor recurrence	
yes	9 (11.25%)
no	71 (88.75%)

Continuous variables are expressed as mean and standard deviation (SD); dichotomous variables are expressed as number of participants and percentage. FIGO: International Federation of Obstetrics and Gynaecology, MMT: Malignant Mixed Müllerian Tumor, MELF: Microcystic, Elongated, and Fragmented, MMRd: Mismatch Repair Deficient; * chemotherapy and/or external beam radiation therapy.

**Table 2:** Fisher’s exact test among [^18^F]FDG PET/MR imaging findings and histological data.

Variable	LN	p53	MMRd	Histotype	Infiltration Pattern	LVSI	Deep MI	Adjuvant Therapy	Relapse
MRI-detected MI	10.214 (0.003 *)	2.348 (0.21)	1.511 (0.85)	1.020 (>0.99)	1.899 (0.29)	5.380 (0.003 *)	30.933 (<0.001 *)	7.511 (0.001 *)	13.667 (0.02 *)
MRI-detected CSI	7.714 (0.007 *)	2.020 (0.45)	0.943 (>0.99)	0.441 (0.57)	4.278 (0.09)	10.476 (0.003 *)	inf (<0.001 *)	11.367 (0.018 *)	4.978 (0.06)
MRI-detected LNs	30.400 (<0.001 *)	4.417 (0.16)	1.667 (0.85)	0.564 (0.57)	2.619 (0.21)	4.333 (0.048 *)	11.724 (0.010 *)	10.000 (0.024 *)	6.629 (0.06)
PET- detected LNs	173.250 (<0.001 *)	2.812 (0.14)	10.357 (0.027 *)	0.802 (0.72)	1.964 (0.31)	12.048 (<0.001 *)	7.661 (0.006 *)	Inf (<0.001 *)	4.978 (0.06)

Table summarizing odds ratio (*p*-values) of Fisher’s exact test describing the association between [^18^F]FDG PET/MR-detected deep myometrial invasion (MI), cervical stroma invasion (CSI), and lymph node metastases (LNs) with investigated outcomes. Statistical significance (*p* < 0.005) is highlighted with *.

## Data Availability

Dataset available on request due to restrictions (e.g., privacy, legal or ethical reasons).

## Supplementary Materials

The following supporting information can be downloaded at https://www.mdpi.com/article/10.3390/cancers18020280/s1: Figure S1: Correlation matrix of [^18^F]FDG PET/MR parameters with clinical and histological data; Table S1: AUC (95% CI) and optimal cut-offs of statistically significant parameters for each investigated outcome.

## Abbreviations

The following abbreviations are used in this manuscript:

SUV	Standardized Uptake Value
MTV	Metabolic Tumor Volume
TLG	Total Lesion Glycolysis
TTV	Total Tumor Volume
TUV	Total Uterine Volume
TVR	Tumor Volume Ratio
Size_AP	Antero-Posterior Diameter
Size_LL	Latero-Lateral Diameter
Size_CC	Cranio-Caudal Diameter
ADC	Apparent Diffusion Coefficient
Ktrans	Transfer Constant
Kep	Efflux Rate
Ve	Extravascular Extracellular Volume
CER	Contrast–Enhancement Ratio
maxSLOPE	Maximum Slope of Increase
