# Supplementary material for: [18F]FDG PET/MRI in Endometrial Cancer: Prospective Evaluation of Preoperative Staging, Molecular Characterization and Prognostic Assessment
Source: Cancers (Basel). 2026 Jan 16;18(2):280. doi: 10.3390/cancers18020280 (PMC12838805; doi:10.3390/cancers18020280)
Supplement: Supplementary file 1 [file cancers-18-00280-s001.zip › cancers-4063089-supplementary.pdf]

[18F]FDG PET/MRI in endometrial cancer: prospective evaluation of preoperative staging, molecular characterization and prognostic assessment

Supplemental Materials

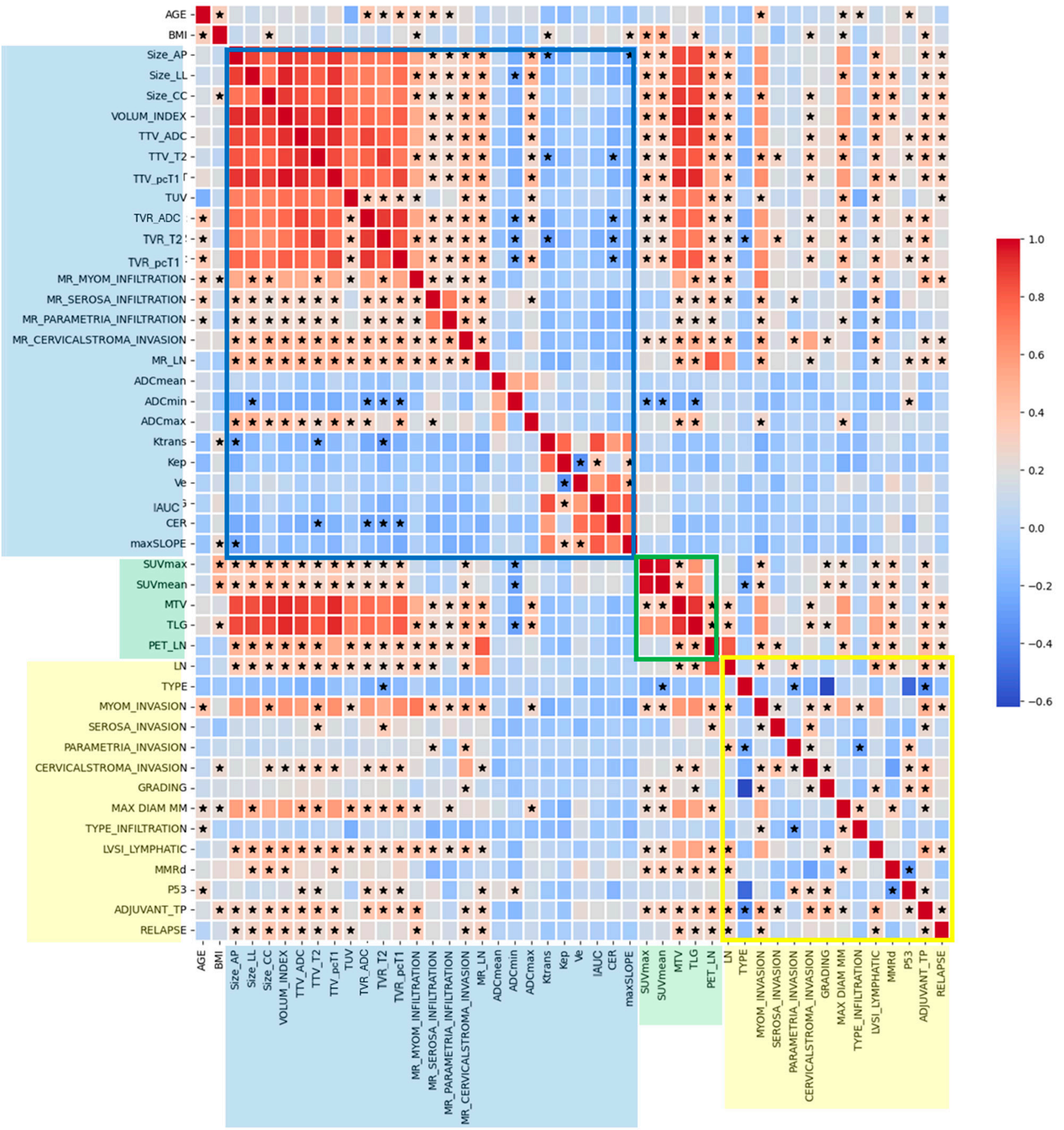

Figure S1. Correlation matrix of [18F]FDG PET/MR parameters with clinical and histological data. Correlation matrix displaying Spearman's correlation among [18F]FDG PET/MR parameters and clinical and histological data. Correlations among [18F]FDG PET, MR, and histology data are highlighted, respectively, in green, blue and yellow. Statistical significance ($p<0.05$) corresponding to each correlation analysis is marked with *. Volumetric parameters are expressed in mL or cm^3; TVR is expressed as percentage (%); ADC values are expressed as 10^{-6} mm^2/s. Size_AP= antero-posterior diameter, Size_LL= latero-lateral diameter, Size_CC= cranio-caudal diameter, TTV= total tumor volume, TUV= total uterine volume, TVR= tumor volume ratio, ADC= apparent diffusion coefficient, Ktrans= transfer constant, Kep= efflux rate, Ve= extravascular extracellular volume, IAUC= integral area under the curve, CER= contrast-enhancement ratio, maxSLOPE= maximum slope of increase,

SUV= standardized uptake value, MTV= metabolic tumor volume, TLG= total lesion glycolysis, LVSI= lymphovascular space invasion, ADJUVANT_TP= adjuvant therapy administration.

Table S1. AUC (95% CI) and optimal cut-offs of statistically significant parameters for each investigated outcome.

Variable	AUC	Lower 95% CI	Upper 95% CI	Optimal cut-off	Sensitivity	Specificity
**Lymph node metastases**						
SUVmax	0.638822	0.500000	0.771795	18.08	0.923077	0.468750
SUVmean	0.646635	0.507172	0.773469	11.35	0.923077	0.500000
MTV	0.752404	0.614744	0.874359	5.59	1.000000	0.468750
TLG	0.782452	0.663399	0.889423	80.40	1.000000	0.546875
Size_AP	0.766026	0.639655	0.882323	18.000000	1.000000	0.433333
Size_LL	0.769231	0.634158	0.879540	34.000000	0.769231	0.700000
Size_CC	0.742308	0.625000	0.850484	35.000000	0.923077	0.566667
VI	0.776923	0.639744	0.885057	15.660000	1.000000	0.483333
TTV_ADC	0.783333	0.672414	0.897541	9.600000	0.923077	0.600000
TTV_T2	0.752564	0.617188	0.859973	8.000000	1.000000	0.466667
**TTV_pcT1**	**0.794872**	**0.667155**	**0.889655**	**9.000000**	**0.923077**	**0.600000**
TUV	0.721795	0.561538	0.865021	87.000000	0.692308	0.750000
TVR_ADC	0.721795	0.567449	0.861905	10.679612	0.923077	0.516667
TVR_T2	0.688462	0.533333	0.826984	42.957746	0.461538	0.883333
TVR_pcT1	0.730769	0.573016	0.882567	39.824561	0.461538	0.950000
**p53abn**						
**AGE**	**0.714718**	**0.568689**	**0.846032**	**61.0**	**0.875**	**0.516129**
TTV_ADC	0.690395	0.538793	0.824859	9.480000	0.800000	0.576271
TTV_T2	0.671186	0.513859	0.807359	10.400000	0.866667	0.542373
**TVR_ADC**	**0.714689**	**0.589815**	**0.837302**	**17.648810**	**0.866667**	**0.593220**
TVR_T2	0.692655	0.552137	0.817187	18.452381	0.866667	0.576271
TVR_pcT1	0.674576	0.522188	0.826389	19.464286	0.733333	0.661017
ADCmean	0.646893	0.501348	0.792857	973.000000	0.800000	0.559322
ADCmin	0.694350	0.500855	0.858333	722.000000	0.533333	0.915254
**MMRd**						
SUVmax	0.694444	0.535104	0.832031	16.44	0.888889	0.600000
SUVmean	0.700000	0.546279	0.834783	11.35	0.833333	0.633333
MTV	0.682407	0.522686	0.841797	4.83	0.888889	0.566667
TLG	0.738889	0.592030	0.875000	75.80	0.833333	0.633333
Size_LL	0.708061	0.554737	0.849359	24.000	0.882353	0.518519
**Size_CC**	**0.745098**	**0.581197**	**0.886710**	**30.000**	**0.941176**	**0.629630**
VI	0.703704	0.531680	0.844017	10.752	0.941176	0.518519
**Histotype**						
SUVmean	0.668783	0.515604	0.816964	13.56	0.733333	0.650794
TTV_T2	0.650000	0.510156	0.778058	8.520000	0.928571	0.466667
TVR_ADC	0.651786	0.519546	0.778409	9.747235	0.928571	0.466667
**TVR_T2**	**0.678571**	**0.560372**	**0.801190**	**9.612142**	**1.000000**	**0.400000**
**Infiltration pattern**						
SUVmean	0.649798	0.504545	0.779545	14.14	0.684211	0.692308
**lymphovascular space invasion**						
SUVmax	0.653872	0.533223	0.769361	17.84	0.81250	0.521739
SUVmean	0.665761	0.539623	0.787143	11.35	0.78125	0.565217
MTV	0.802310	0.713315	0.897059	7.47	0.84375	0.717391

	**TLG**	**0.819973**	**0.718954**	**0.911111**	**114.20**	**0.75000**	**0.826087**
	Size_AP	0.734375	0.609860	0.849084	19.000000	0.87500	0.595238
	Size_LL	0.745164	0.615530	0.858918	31.000000	0.68750	0.785714
	Size_CC	0.763393	0.640285	0.862490	29.000000	0.90625	0.523810
	VI	0.776042	0.655584	0.879121	25.947000	0.71875	0.761905
	TTV_ADC	0.754836	0.626755	0.854532	7.000000	0.87500	0.619048
	TTV_T2	0.710193	0.583851	0.831871	9.400000	0.81250	0.619048
	TTV_pcT1	0.771577	0.656319	0.880460	7.450000	0.87500	0.595238
	TUV	0.691592	0.568558	0.805698	62.000000	0.71875	0.619048
	TVR_ADC	0.704241	0.583150	0.813747	7.970027	0.90625	0.547619
	TVR_T2	0.663690	0.536029	0.793792	10.555556	0.84375	0.547619
	TVR_pcT1	0.719494	0.596491	0.842461	8.737864	0.87500	0.571429
**Myometrial Invasion**							
	AGE	0.653846	0.527303	0.769560	70.00	0.487179	0.846154
	SUVmax	0.640039	0.520105	0.765943	18.93	0.717949	0.564103
	MTV	0.788626	0.678312	0.878788	7.55	0.769231	0.769231
	**TLG**	**0.797830**	**0.685050**	**0.889474**	**119.40**	**0.692308**	**0.897436**
	Size_AP	0.772894	0.664080	0.874344	23.000000	0.692308	0.800000
	Size_LL	0.780586	0.671293	0.885045	27.000000	0.769231	0.742857
	Size_CC	0.733333	0.607353	0.851562	43.000000	0.589744	0.857143
	VI	0.787546	0.675758	0.876969	28.971000	0.615385	0.914286
	TTV_ADC	0.754212	0.639518	0.858918	15.000000	0.538462	0.914286
	TTV_T2	0.721612	0.596691	0.830007	17.000000	0.564103	0.828571
	TTV_pcT1	0.794872	0.693382	0.888605	8.190000	0.769231	0.714286
	TUV	0.652015	0.517909	0.771324	81.000000	0.512821	0.800000
	TVR_ADC	0.745421	0.634066	0.852640	8.982036	0.846154	0.628571
	TVR_T2	0.714286	0.592593	0.827457	10.555556	0.820513	0.600000
	TVR_pcT1	0.783883	0.677137	0.879474	7.577197	0.897436	0.600000
	ADCmax	0.652381	0.527473	0.772321	1550.000000	0.820513	0.485714
**Need for adjuvant therapy**							
	BMI	0.651786	0.523569	0.774733	24.349609	0.857143	0.500000
	SUVmax	0.676587	0.539439	0.803419	17.840000	0.809524	0.611111
	SUVmean	0.683862	0.552926	0.798671	12.340000	0.738095	0.666667
	MTV	0.686177	0.554233	0.813542	5.590000	0.809524	0.611111
	TLG	0.703373	0.579514	0.811821	60.900000	0.785714	0.611111
	Size_AP	0.678123	0.549150	0.802632	19.000000	0.780488	0.606061
	Size_LL	0.664819	0.536585	0.794118	29.000000	0.634146	0.727273
	Size_CC	0.696970	0.572727	0.807664	29.000000	0.804878	0.515152
	VI	0.701404	0.573143	0.825321	15.660000	0.804878	0.636364
	TTV_ADC	0.713969	0.593168	0.826820	7.000000	0.780488	0.636364
	**TTV_T2**	**0.735033**	**0.619177**	**0.841544**	**7.000000**	**0.853659**	**0.575758**
	TTV_pcT1	0.688840	0.566667	0.805701	8.000000	0.756098	0.636364
	TVR_ADC	0.714339	0.591017	0.825573	7.813411	0.853659	0.575758
	TVR_T2	0.733925	0.604167	0.837363	10.555556	0.804878	0.606061
	TVR_pcT1	0.682188	0.559211	0.800450	8.737864	0.780488	0.575758
	Ve	0.631190	0.500368	0.760985	0.579000	0.487805	0.818182
**Tumor recurrence**							
	**MTV**	**0.818035**	**0.701207**	**0.912162**	**13.49**	**0.888889**	**0.768116**
	TLG	0.739130	0.565807	0.903421	201.30	0.666667	0.782609
	Size_AP	0.725641	0.555871	0.882729	23.000000	0.777778	0.584615
	Size_LL	0.756410	0.579957	0.899061	31.000000	0.888889	0.646154

	Size_CC	0.815385	0.679487	0.924786	43.000000	0.888889	0.692308
	VOLUM_INDEX	0.788034	0.653571	0.903571	25.947000	0.888889	0.615385
	TTV_ADC	0.757265	0.590618	0.885928	12.200000	0.888889	0.661538
	TTV_T2	0.722222	0.563725	0.868545	17.900000	0.777778	0.692308
	TTV_T1_POST	0.775214	0.618750	0.907246	17.000000	0.777778	0.769231
	TUV	0.710256	0.517157	0.881250	66.000000	0.777778	0.584615
	TVR1adc	0.692308	0.500000	0.871429	7.166948	1.000000	0.323077
	TVR1post	0.694017	0.512821	0.873106	21.696970	0.666667	0.707692

Performance metrics from the Receiver Operating Characteristic (ROC) analysis evaluating the predictive capacity of PET, MRI, and clinical parameters for the different investigated outcomes. For each parameter, the table includes the Area Under the Curve (AUC), the 95% Confidence Interval-CI (Lower 95% CI - Upper 95% CI), the optimal threshold and respective sensitivity and specificity. The parameter providing the best performance is highlighted in bold. Only statistically significant results are included. Volumetric parameters are expressed in mL or cm^3; TVR is expressed as percentage (%); ADC values are expressed as 10^{-6} mm^2/s. SUV= standardized uptake value, MTV= metabolic tumor volume, TLG= total lesion glycolysis, Size_AP= antero-posterior diameter, Size_LL= latero-lateral diameter, Size_CC= cranio-caudal diameter, VI= volume index, TTV= total tumor volume, TUV= total uterine volume, TVR= tumor volume ratio, Ve= extravascular extracellular volume, ADC= apparent diffusion coefficient.
